# Acute tetraparesis secondary to bilateral precentral gyral cerebral ischemia: a case report

**DOI:** 10.1186/1752-1947-7-61

**Published:** 2013-03-08

**Authors:** Christian Geis, Ignaz Gunreben, Christoph Kleinschnitz

**Affiliations:** 1 Department of Neurology, University of Würzburg, Josef-Schneider-Str. 11, Wuerzburg 97080, Germany; 2 Hans Berger Department of Neurology, Jena University Hospital, Erlanger Allee 101, Jena 07747, Germany

## Abstract

**Introduction:**

Sudden tetraparesis represents a neurological emergency and is most often caused by traumatic spinal cord injury, spinal epidural bleeding or brainstem ischemia and less frequently by medial disc herniation or spinal ischemia.

**Case presentation:**

Here we report the rare case of an 82-year-old Caucasian man who developed severe tetraparesis four days after radical cystoprostatectomy. An emergency diagnostic study for spinal cord affection was normal. Brain magnetic resonance imaging revealed acute bilateral ischemic strokes in the precentral gyri as the underlying cause.

**Conclusions:**

This case report underlines the need to also consider unusual causes of tetraparesis in an emergency situation apart from spinal cord or brain stem injury in order not to leave severe symptomatology unclear and possibly miss therapeutic options.

## Introduction

Sudden tetraparesis is a neurological emergency and is most often caused by traumatic spinal cord injury, spinal epidural bleeding or brainstem ischemia and less frequently by medial disc herniation or spinal ischemia. We present an interesting case in which sudden tetraparesis was caused by bilateral precentral gyral ischemia.

## Case presentation

An 82-year-old Caucasian man was admitted to our urology department for radical cystoprostatectomy because of prostate cancer.

During the night of the fourth day post-surgery the patient suddenly developed painless severe tetraparesis (Medical Research Council grade 2/5 of proximal muscles on both arms and 0/5 of both legs) without involvement of cranial nerves or impaired consciousness. There were no signs of sensory deficits. He did not present with any cerebellar symptoms. Further clinical examination revealed brisk tendon reflexes and positive pyramidal signs on both sides. The clinical presentation was highly suggestive for spinal cord affection, for example by abrupt compression, subdural bleeding or ischemia. There was no trauma or fall shortly before the onset of the neurological deficits. For analgesic treatment, a peridural catheter had been implanted after surgery which had been removed without any complications the day before.

**Figure 1:**
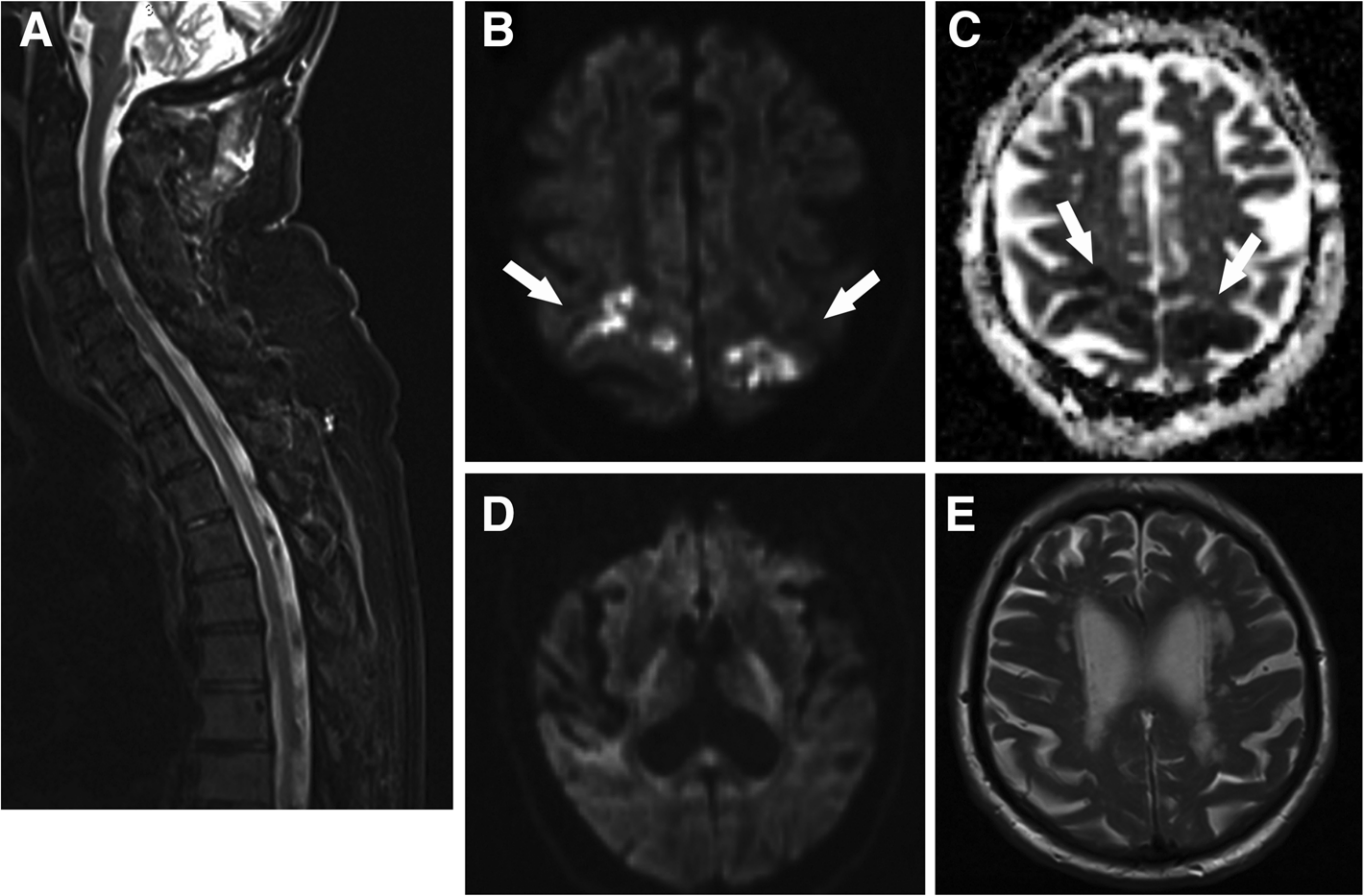
**Bilateral acute ischemia in the motor cortex area. (A)** Spinal cord magnetic resonance imaging (MRI) was normal and showed no signs of compression or ischemia. Of note, acute infarction, for example of the anterior spinal artery may be undetected in early spinal MRI. **(B** and **C)** Diffusion **(B)** and apparent diffusion coefficient-weighted **(C)** brain MRI revealed acute infarctions in the precentral gyrus on both sides (medial and parietal parts). **(D)** At the level of the internal capsule no pathological diffusion restrictions were found. **(E)** In contrast to diffusion, T2-weighted images did not show any signs of tissue demarcation confirming the acuteness of stroke onset.

For an emergency magnetic resonance imaging (MRI) diagnostic, metal brackets in the subcutaneous tissue placed after surgery had to be removed at night. Surprisingly, spinal MRI did not reveal spinal cord compression or signs pointing towards spinal cord ischemia (Figure 1A). By contrast, cranial MRI imaging showed bilateral acute infarctions in the median precentral gyrus (Figure 1B and C; arrows), whereas the internal capsule was unaffected on both sides (Figure 1D). The T2-weighted images at the level of primary motor cortex did not yet present with abnormal signal, thus confirming acute ischemia consistent with the time course of symptom occurrence (Figure 1E). The precentral area of the primary motor cortex is supplied by the anterior cerebral artery (medial one third of the precentral gyrus) and the middle cerebral artery (lateral two thirds) on either side [1].

In the following days, paresis in both arms resolved but the legs remained paraplegic. Further diagnostic workup including carotid duplex sonography, 24-hour electrocardiogram (ECG) and transesophageal echocardiography failed to identify the underlying cause of stroke. Hypercoaguability due to prostate cancer may have been contributed to cerebral ischemia. Routine coagulation parameters were normal, and more extensive coagulation diagnostics were not performed in the acute phase of the cerebral ischemia. Due to the simultaneous bilateral territorial infarctions in at least two independent vascular territories, the absence of macroangiopathy, and the preceding extensive surgery with narcosis we considered a cardiac embolic origin. Therefore the patient was placed on full anticoagulant dose of low-molecular weight heparin for embolic stroke prophylaxis. Early prophylactic treatment with antiplatelet drugs was not possible according to current guidelines because of extensive surgical intervention four days before stroke onset. In consent with the surgeons, we decided in this case to use low-molecular weight heparin as an early prophylactic treatment. After neurological rehabilitation we started a prophylactic treatment with acetylsalicylic acid because repeated 24-hour ECG could not identify a paroxysmal atrial fibrillation.

## Discussion

Sudden tetraparesis represents a neurological emergency. In the majority of cases the tetraparesis is caused by acute mechanical spinal cord affection, for example spinal cord contusion or medial disc herniation [2,3], or by vascular spinal cord pathology, for example spinal epidural bleeding [4], spinal or brainstem ischemia [5].

Here we describe a surprising and unusual cause of acute tetraparesis; in this special case, thrombolytic therapy was not allowed because of preceding surgery. However, immediate diagnostic steps had to be prompted to rule out progressive and potentially reversible spinal cord injury such as mechanical compression. This case report underlines the need to consider unusual causes of tetraparesis in an emergency situation apart from spinal cord or brain stem injury. Although not possible in our case, thrombolytic therapy may also serve as a potential beneficial treatment in bilateral central ischemic stroke within the time window of 4.5 hours if defined criteria are met. Therefore, timely diagnostic steps have to be undertaken and one should also consider a supratentorial origin of sudden tetraparesis [6,7].

## Conclusions

Although unusual, acute tetraparesis may be caused by bilateral precentral gyral cerebral ischemia.

## Consent

Written informed consent was obtained from the patient for publication of this case report and accompanying images. A copy of the written consent is available for review by the Editor-in-Chief of this journal.

## Abbreviations

ECG: Electrocardiogram; MRI: Magnetic resonance imaging.

## Competing interests

The authors declare that they have no competing interests.

## Authors’ contributions

CG designed the study and wrote the manuscript; IG collected data and drafted the manuscript; CK wrote and revised the manuscript. All authors read and approved the final manuscript.
